# Triglyceride-glucose index as a potential predictor of major adverse cardiovascular and cerebrovascular events in patients with coronary heart disease complicated with depression

**DOI:** 10.3389/fendo.2024.1416530

**Published:** 2024-06-26

**Authors:** Weizhe Zhao, Junqing Wang, Dong Chen, Wanli Ding, Jiqiu Hou, YiWei Gui, Yunlin Liu, Ruiyi Li, Xiang Liu, Zhiqi Sun, Haibin Zhao

**Affiliations:** ^1^ The Dongfang Hospital of Beijing University of Chinese Medicine, Beijing, China; ^2^ Department of Oncology, Beijing Hospital of Traditional Chinese Medicine, Capital Medical University, Beijing, China

**Keywords:** triglyceride-glucose index, predictor, MACCE, coronary heart disease, depression

## Abstract

**Background:**

Triglyceride-glucose (TyG) index is a surrogate marker of insulin resistance and metabolic abnormalities, which is closely related to the prognosis of a variety of diseases. Patients with both CHD and depression have a higher risk of major adverse cardiovascular and cerebrovascular events (MACCE) and worse outcome. TyG index may be able to predict the adverse prognosis of this special population.

**Methods:**

The retrospective cohort study involved 596 patients with both CHD and depression between June 2013 and December 2023. The primary outcome endpoint was the occurrence of MACCE, including all-cause death, stroke, MI and emergent coronary revascularization. The receiver operating characteristic (ROC) curve, Cox regression analysis, Kaplan-Meier survival analysis, and restricted cubic spline (RCS) analysis were used to assess the correlation between TyG index and MACCE risk of in patients with CHD complicated with depression.

**Results:**

With a median follow-up of 31 (15–62) months, MACCE occurred in 281(47.15%) patients. The area under the ROC curve of TyG index predicting the risk of MACCE was 0.765(0.726–0.804) (*P*<0.01). Patients in the high TyG index group(69.73%) had a significantly higher risk of developing MACCE than those in the low TyG index group(23.63%) (*P*<0.01). The multifactorial RCS model showed a nonlinear correlation (nonlinear *P*<0.01, overall *P*<0.01), with a critical value of 8.80 for the TyG index to predict the occurrence of MACCE. The TyG index was able to further improve the predictive accuracy of MACCE.

**Conclusions:**

TyG index is a potential predictor of the risk of MACCE in patients with CHD complicated with depression.

## Background

The latest epidemiological report by the World Health Organization shows that cardiovascular disease (CVD) is the primary cause of death globally, contributing to approximately 32% of global mortality (1). Ischemic heart disease contributes to a large proportion of these deaths, accounting for approximately 16% of global deaths (2). Depression is a disorder characterized by depressed mood (e.g., sadness, irritability, emptiness, or loss of pleasure), frequently accompanied by additional cognitive, behavioral, or neurovegetative symptoms that have a notable impact on patient’s functioning (3). Depression is prevalent among patients with CVD (4, 5), with approximately 20–30% of patients with coronary heart disease (CHD) experiencing depression (6, 7). Studies have demonstrated that, in patients with CHD, depression not only significantly affects patient’s quality of life (5) but also increases the risk of all-cause death, cardiac-related death, and new cardiac events by 2.3-fold, 2.7-fold, and 1.6-fold, respectively (4, 8, 9).

Encouragingly, 86% of cardiovascular (CV) deaths can be prevented by addressing behavioral risk factors (10). Therefore, there is an urgent need to identify patients at high risk for CHD comorbid with depression by exploring new predictors, allowing for the implementation of early interventions to reduce the incidence of adverse CV events. In recent years, several studies have demonstrated that insulin resistance (IR) not only has a significant impact on the development of metabolic syndrome (MetS) and type 2 diabetes mellitus (T2DM), but is also closely related to the onset of CVD and atherosclerotic disease (11–13). In addition, IR can also adversely affect the prognosis of patients with psychiatric disorders, such as anxiety and depression, by affecting neuronal and synaptic activity and exacerbating neuroinflammation (14–16). Therefore, IR may be a key factor affecting the prognosis of patients with CHD complicated with depression.

The triglyceride-glucose (TyG) index is a representative marker of IR and is calculated as follows: ln[fasting blood glucose × fasting triglyceride/2]. Despite its lower accuracy for assessing IR compared with that of the gold standard high insulin normoglycemic clamp, it is still widely used because of its simplicity, reliability, and ready applicability (17, 18).

Previous studies have demonstrated that the TyG index score is not only significantly associated with T2DM, MetS, and atherosclerosis but also served as an independent risk factor for the occurrence of major adverse cardiovascular and cerebrovascular events (MACCE) in CHD, regardless of the patient having T2DM (19, 20). TyG levels are closely related to the occurrence and progression of depression (21, 22). Therefore, TyG may be an important prognostic indicator in patients with CHD complicated with depression.

However, no studies have evaluated the prognostic significance of the TyG index score in patients with CHD complicated with depression. Therefore, this study aimed to explore the predictive value of the TyG index score for MACCE in patients with both CHD and depression.

## Methods

### Study participants

**Figure 1:**
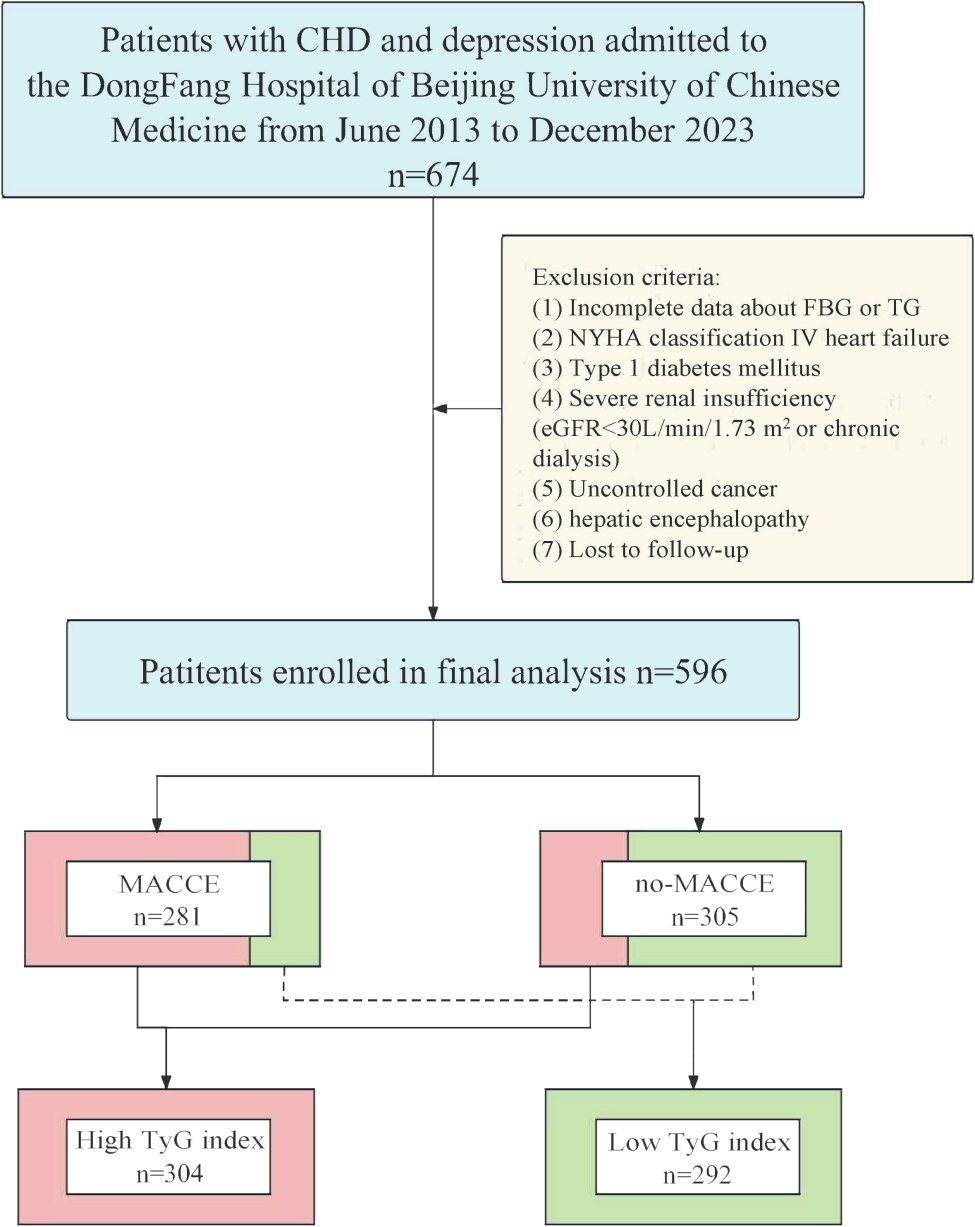
Flow chart of patients screening and grouping. CHD, coronary artery disease; FBG, fasting blood glucose; NYHA, New York Heart Association; TG, triglyceride; eGFR, estimated glomerular filtration rate; MACCE, major adverse cardiovascular and cerebrovascular event; TyG, triglyceride-glucose.

This single-center, retrospective, cohort study involved 596 patients with comorbid CHD and depression who sought treatment at the Dongfang Hospital of Beijing University of Chinese Medicine from June 2013 to December 2023. The following criteria were used for inclusion: (1) individuals aged 18 years or older; (2) Patients with a diagnosis of CHD; (3) Patients meeting the diagnosis of major depressive disorder according to the Diagnostic and Statistical Manual of Mental Disorders, Fifth Edition (DSM-5). CHD was defined based on meeting at least one of the criteria (23): (1) Percutaneous coronary angiography or computed tomographic angiography revealed ≥ 50% stenosis in at least one coronary artery trunk or primary branch; (2) Presence of typical symptoms of exertional angina, accompanied by a positive stress test (electrocardiogram stress test, stress echocardiography, or nuclide myocardial stress imaging); (3) A documented history of myocardial infarction (MI); (4) Previous diagnosis of unstable. angina pectoris, characterized by typical ischemic chest pain, electrocardiogram changes, and elevated markers of muscle damage, or dynamic ST segment changes during ischemic episodes, or confirmation of severe lesions through coronary angiography leading to symptomatic manifestations. The definition of depression refers to the DSM-5 diagnostic criteria for major depressive disorder (24). The exclusion criteria included: (1) Incomplete data about fasting blood glucose (FBG) or triglyceride (TG); (2) New York Heart Association(NYHA) classification IV heart failure; (3) Type1 diabetes mellitus;(4) Severe renal insufficiency (eGFR<30mL/min/1.73 m^2^ or chronic dialysis); (5) Uncontrolled cancer; (6) Hepatic encephalopathy; (7) Lost to follow-up (**Figure 1**).

Ultimately, a total of 596 patients were included in the analysis. The patients were categorized into a MACCE (n=281) or no-MACCE (n=315) group according to the occurrence of MACCE during follow-up. After conducting receiver operating characteristic (ROC) curve analysis to identify the ideal threshold for the TyG index, patients were divided into the low group (n=292) and the high group (n=304) based on this categorization.

This study follows the principles of the Helsinki Declaration and has been approved by the Clinical Research Ethics Committee of the Dongfang Hospital of Beijing University of Chinese Medicine. All personal information regarding the patients’ identities was de-identified.

### Data collection and definitions

Data regarding demographics; vital signs; smoking (smoking was described as a total of more than 100 cigarettes in a patient’s lifetime, regardless of whether they are currently quitting or not (25)); laboratory measurements (total cholesterol [TC], triglyceride [TG], high-density lipoprotein cholesterol [HDL-C], low-density lipoprotein cholesterol [LDL-C], fasting blood glucose [FBG], and glycosylated hemoglobin A1c [HbA1c]); case history; a family history of CVDs; and use of medications. Body mass index (BMI) was calculated as weight (kg)/[height (m)]^2^ (26).

### Follow-up and endpoints

All patients were followed up via telephone or outpatient visits by professionally trained personnel. The follow-up lasted until December 2023, unless detachment or death occurred. The primary endpoint of this study was the composite endpoint of MACCE, encompassing the occurrence of all-cause death, stroke, MI, or emergent coronary revascularization during the follow-up period (based on the first secondary endpoint event after discharge or the most severe event if multiple endpoint events occurred simultaneously, with the events prioritized as follows: all-cause death > stroke > MI > emergent coronary revascularization). End-point events were determined independently by two cardiovascular specialists who were not aware of the patient’s TyG index scores or other baseline information. When there was disagreement regarding the determination of the endpoint event, a third expert was consulted, and a joint decision was made after discussion.

### Statistical analysis

Statistical analyses in this study were calculated with SPSS software (version 26.0) and R software (version 4.2.3). The P-value <0.05 indicated a statistically significant difference. Continuous variables, including age, BMI and blood pressure were expressed as mean ± standard deviation (SD); continuous variables that did not follow a normal distribution, including TC, LDL-C, HDL-C, FBG, and HbA1c, were presented as median and interquartile range. We used the independent samples t-test or Wilcoxon rank-sum test to compare continuous variables between the groups. The categorical variables, including sex, smoking, case history, and use of medications, are presented as frequencies (percentages). We compared categorical variables between groups by using Pearson’s chi-square test or Fisher’s exact test.

We conducted a systematic analysis of the relationship between the TyG index and MACCE in patients with depression and CHD. ROC curve analysis was employed to ascertain the ideal threshold value of TyG index in forecasting the occurrence of MACCE, and additionally evaluated the added discriminatory power of the TyG index group beyond the initial risk model. The Kaplan-Meier survival analyses were conducted to evaluate the incidence of endpoint events in both cohorts.

In order to determine if the TyG index score could function as an independent predictor of the incidence of MACCE, we performed univariate and multivariate Cox regression analyses, presenting results as hazard ratios (HR) and 95% confidence intervals (CI). In addition, four different Cox proportional risk models were developed to identify independent risk factors for MACCE in each model. Model 1 was adjusted for age, male sex, BMI, systolic blood pressure, diastolic blood pressure, and smoking; On the basis of Model 1, Model 2 was adjusted for hypertension, T2DM, dyslipidemia, prior CVDs, prior PCI, prior stroke and a family history of CVDs; Model 3 adds variables from clinical tests to Model 2: TC, LDL-C, HDL-C, and HbA1c; and Model 4 was adjusted for the variables included in Model 3 and the use of antiplatelet medication, angiotensin-converting enzyme inhibitor/angiotensin receptor blocker (ACEI/ARB), calcium channel blocker (CCB), β-Blocker, antidiabetic agents, statins, antidepressants, and benzodiazepines. Furthermore, Model 4 adjustments were utilized to construct restricted cubic spline (RCS) curves, demonstrating the nonlinear or linear correlation between the TyG index score and MACCE.

In addition, subgroup analyses were conducted to explore the consistency of the TyG index score’s predictive efficacy for MACCE among various subgroups. Besides, we employed the integrated discrimination improvement(IDI), net reclassification improvement (NRI) and concordance index (C-index) to examine the incremental benefit of the TyG index in forecasting MACCE.

## Results

### Baseline information

A total of 596 patients were ultimately involved in this study. The mean age was 71.71 ± 9.36 years, of which 212 (35.6%) were male. The baseline characteristics of the total population, grouped according to whether MACCE occurred or not, are shown in **Table 1**. The TyG index of patients with MACCE(9.10, 8.80–9.47) was significantly higher than that of patients without MACCE (8.48, 8.19–8.86) (*P*<0.01). In addition, patients in the MACCE group demonstrated substantially elevated levels of BMI, TC, TG, LDL-C, FBG, and HbA1c compared to those in the no-MACCE group. More patients in the MACCE group had diabetes as well as previous cerebral infarction than those in the no-MACCE group. Meanwhile, the proportion of patients in the MACCE group receiving ACEI/ARB, hypoglycemic agents, and statins was substantially higher than that in the no-MACCE group. The proportion of patients receiving antidepressants in MACCE group was similar to that in non MACCE group.

**Table 1:** Baseline characteristics of population divided by MACCE-related situation.

Characteristics	Overall (n=596)	MACCE (n=281)	No-MACCE (n=315)	*P* value
Age (years)	71.71 ± 9.36	72.46 ± 9.76	71.05 ± 8.96	0.068
Male (n, %)	212(35.6%)	102(36.3%)	110(34.9%)	0.726
BMI (kg/m^2^)	24.53 ± 3.64	24.87 ± 3.93	24.22 ± 3.33	0.031
Smoking (n, %)	147(24.66%)	77(27.40%)	70(22.22%)	0.143
SBP (mmHg)	133.56 ± 19.16	133.76 ± 19.02	133.37 ± 19.31	0.804
DBP (mmHg)	75.62 ± 11.21	75.03 ± 10.78	76.14 ± 11.58	0.229
Laboratory variables				
TC (mmol/L)	3.86(3.26~4.57)	4.05(3.34~4.86)	3.74(3.17~4.34)	<0.01
TG(mmol/L)	1.31(0.96~1.92)	1.63(1.20~2.16)	1.10(0.86~1.55)	<0.01
HDL-C(mmol/L)	1.22(1.00~1.47)	1.20(0.98~1.45)	1.23(1.02~1.49)	0.127
LDL-C(mmol/L)	2.26(1.72~2.87)	2.37(1.85~3.00)	2.20(1.66~2.73)	<0.01
FBG(mmol/L)	5.82(5.05~7.63)	6.59(5.34~8.81)	5.38(4.90~6.40)	<0.01
HbA1c(%)	5.80(5.40~6.80)	6.00(5.40~7.25)	5.60(5.30~6.40)	<0.01
TyG index	8.80(8.36~9.26)	9.10(8.80~9.47)	8.48(8.19~8.86)	<0.01
Case history				
Hypertension (n, %)	467(78.36%)	223(79.36%)	244(77.46%)	0.574
T2DM (n, %)	292(49.00%)	163(58.00%)	129(40.95%)	<0.01
Dyslipidemia (n, %)	388(65.10%)	184(65.48%)	204(64.76%)	0.854
Prior CVDs (n, %)	506(84.90%)	241(85.77%)	265(84.13%)	0.577
Prior PCI (n, %)	161(27.01%)	79(28.11%)	82(26.03%)	0.568
Prior stroke (n, %)	264(44.30%)	144(51.25%)	120(38.10%)	<0.01
Family history of CVDs (n, %)	183(30.70%)	78(27.76%)	105(33.33%)	0.141
Medications				
Antiplatelet medication (n, %)	565(94.80%)	262(93.24%)	303(96.19%)	0.105
ACEI/ARB (n, %)	262(43.96%)	155(55.16%)	107(33.97%)	<0.01
CCB (n, %)	334(56.04%)	155(55.16%)	179(56.83%)	0.683
β-Blocker (n, %)	351(58.89%)	173(61.57%)	178(56.51%)	0.210
Antidiabetic agents (n, %)	253(42.45%)	151(53.74%)	102(32.38%)	<0.01
Statins (n, %)	516(86.58%)	263(93.59%)	253(80.32%)	<0.01
Antidepressants (n, %)	405(67.95%)	189(67.26%)	216(68.57%)	0.732
Benzodiazepines (n, %)	214(35.91%)	102(36.30%)	112(35.56%)	0.850

MACCE, major adverse cardiovascular and cerebrovascular events; BMI, body mass index; SBP, systolic blood pressure; DBP, diastolic blood pressure; TC, total cholesterol; TG, triglyceride; LDL-C, low-density lipoprotein cholesterol; HDL-C, high-density lipoprotein cholesterol; FBG, fasting blood glucose; HbA1c, glycosylated hemoglobin; TyG, triglyceride-glucose;T2DM, diabetes mellitus type 2; CVD, cardiovascular disease; PCI, percutaneous coronary intervention; ACEI, angiotensin converting enzyme inhibitor; ARB, angiotensin receptor blocker; CCB, calcium channel blocker.

**Figure 2:**
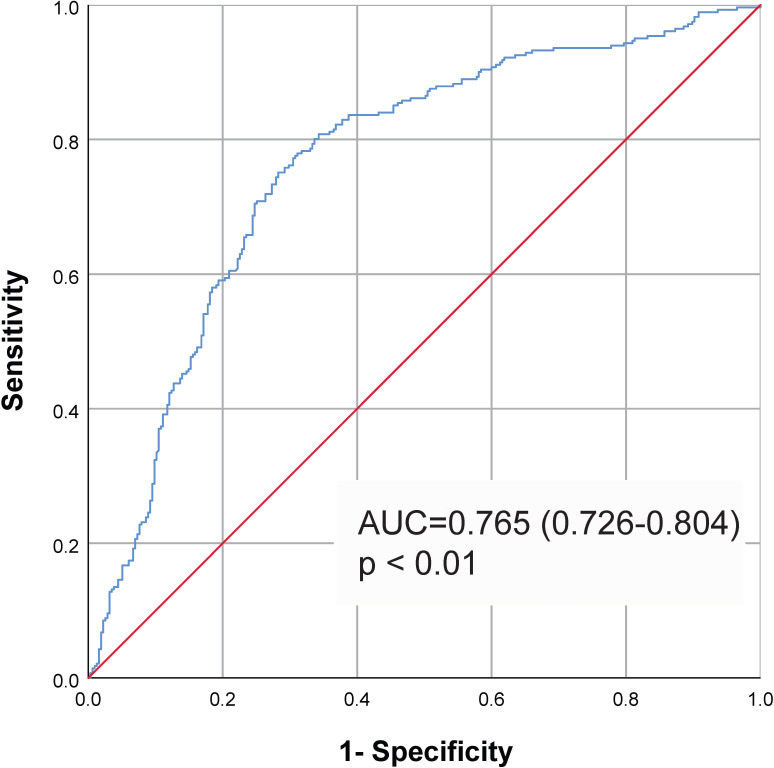
ROC curve to assess the diagnostic performance of the TyG index for MACCE. Cut-off value 8.80, AUC=0.765, 95% CI (0.726–0.804), *P*<0.01. Sensitivity: 75.4%; Specificity: 70.8%. ROC, receiver operating characteristic; AUC, area under the curve; TyG, triglyceride-glucose; MACCE, major adverse cardiovascular and cerebrovascular event.

By ROC curve analysis (**Figure 2**), we found that the TyG index predicted MACCE with an area under the curve of 0.765 (0.726–0.804, *P*<0.01). It was determined that 8.80 was the best cut-off value for the TyG index in predicting MACCE (sensitivity: 75.4%; specificity: 70.8%). The participants was divided into the low group and the high group based on the best cut-off value of the TyG index. The baseline characteristics of the different groups are shown in **Table 2**. We observed that the BMI of patients in the high TyG group was significantly higher than that of patients in the low TyG index group. TG, TC, LDL-C, FBG, and HbA1c levels were significantly higher, and HDL-C was significantly lower in the high TyG index group. In addition, more patients in the high TyG index group were treated with ACEI/ARB, hypoglycemic agents, and statins.

**Table 2:** Baseline characteristics of population divided by TyG index-related situation.

Characteristics	Overall (n=596)	High TyG index (n=304)	Low TyG index (n=292)	*P* value
Age (years)	71.71 ± 9.36	71.19 ± 9.67	72.26 ± 9.01	0.161
Male, n (%)	212(35.57%)	101(33.22%)	111(38.01%)	0.222
BMI (kg/m^2^)	24.53 ± 3.64	25.30 ± 3.72	23.72 ± 3.37	<0.01
Smoking n (%)	147(24.66%)	73(25.00%)	74(24.34%)	0.852
SBP (mmHg)	133.56 ± 19.16	134.10 ± 19.13	133.00 ± 19.20	0.486
DBP (mmHg)	75.62 ± 11.21	75.46 ± 11.20	75.78 ± 11.25	0.733
Laboratory variables				
TC (mmol/L)	3.86(3.26~4.57)	4.10(3.41~4.98)	3.64(3.13~4.21)	<0.01
TG(mmol/L)	1.31(0.96~1.92)	1.91(1.45~2.33)	0.97(0.80~1.18)	<0.01
HDL-C(mmol/L)	1.22(1.00~1.47)	1.15(0.95~1.36)	1.29(1.06~1.62)	<0.01
LDL-C(mmol/L)	2.26(1.72~2.87)	2.43(1.91~3.13)	2.13(1.65~2.64)	<0.01
FBG(mmol/L)	5.82(5.05~7.63)	7.14(5.68~9.31)	5.27(4.79~5.84)	<0.01
HbA1c(%)	5.80(5.40~6.80)	6.30(5.50~7.50)	5.50(5.30~6.00)	<0.01
TyG index	8.80(8.36~9.26)	9.25(8.98~9.58)	8.34(8.12~8.55)	<0.01
Case history				
Hypertension n (%)	467(78.36%)	239(78.62%)	228(78.08%)	0.874
T2DM n (%)	292(48.99%)	196(64.47%)	96(32.88%)	<0.01
Dyslipidemia n (%)	388(65.10%)	204(67.11%)	184(63.01%)	0.295
Prior CVDs n (%)	506(84.90%)	250(82.24%)	256(87.67%)	0.064
Prior PCI n (%)	161(27.01%)	89(29.28%)	72(24.66%)	0.204
Prior stroke n (%)	264(44.30%)	144(47.37%)	120(41.10%)	0.123
Family history of CVDs n (%)	183(30.70%)	98(32.24%)	85(29.11%)	0.408
Medications				
Antiplatelet medication n (%)	565(94.80%)	285(93.75%)	280(95.89%)	0.239
ACEI/ARB n (%)	262(43.96%)	162(53.29%)	100(34.25%)	<0.01
CCB n (%)	334(56.04%)	175(57.57%)	159(54.45%)	0.444
β-Blocker n (%)	351(58.89%)	181(59.54%)	170(58.22%)	0.743
Antidiabetic agents n (%)	253(42.45%)	183(60.20%)	70(23.97%)	<0.01
Statins n (%)	516(86.58%)	277(91.12%)	239(81.85%)	<0.01
Antidepressants n (%)	405(67.95%)	198(65.13%)	207(70.89%)	0.132
Benzodiazepines n (%)	214(35.91%)	109(35.86%)	105(35.96%)	0.979

BMI, body mass index; SBP, systolic blood pressure; DBP, diastolic blood pressure; TC, total cholesterol; TG, triglyceride; LDL-C, low-density lipoprotein cholesterol; HDL-C, high-density lipoprotein cholesterol; FBG, fasting blood glucose; HbA1c, glycosylated hemoglobin; TyG, triglyceride-glucose;T2DM, diabetes mellitus type 2; CVD, cardiovascular disease; PCI, percutaneous coronary intervention; ACEI, angiotensin converting enzyme inhibitor; ARB, angiotensin receptor blocker; CCB, calcium channel blocker.

### Association of TyG Index with cardiovascular risk factors

Correlation analysis showed that the TyG index was associated with several cardiovascular risk factors. As shown in Additional file 1: **Supplementary Table S1**, TyG index was positively associated with BMI (*P*<0.01), TC (*P*<0.01), LDL-C (*P*<0.01), HbA1c (*P*<0.01), and history of T2DM (*P*<0.01), whereas it was negatively correlated with age (*P*=0.046), male sex (*P*=0.025), and HDL-C (*P*<0.01).

### Predictive value of the TyG index for the occurrence of MACCE in patients with CHD and depression

During a median follow-up of 31 (15–62) months, 281 (47.15%) patients developed MACCE, including 111 (18.62%) all-cause deaths, 67 (11.24%) stroke, 63 (10.57%) MI, and 40 (6.71%) emergent coronary revascularization (**Table 3**). The incidence of MACCE in patients with high TyG index is significantly higher than that in patients with low TyG index, both in the overall incidence of MACCE and the incidence of each subgroup. The overall incidence of MACCE in the high TyG group (69.73%) was considerably higher than that in the low TyG group (23.63%). Moreover, compared with the low TyG group (13.36%), the high TyG group showed a higher incidence of all-cause death (23.68%). The incidence of stroke in the high TyG group (18.42%) was also significantly higher than that in the low TyG group (3.77%). Similarly, 16.12% of patients in the high TyG group had MI, which was higher than that in the low TyG group (4.79%); the incidence of emergent coronary revascularization was 11.51% in the high TyG group, which was higher than that in the low TyG group (1.71%). All the differences were statistically significant (*P*<0.01) (**Table 3**).

**Table 3:** Comparison of endpoint events between high and low TyG index groups.

Variable, n (%)	Total	Low TyG index	High TyG index	*P* value
	(n=596)	(n=292)	(n=304)	
**MACCE**	281(47.15%)	69(23.63%)	212(69.73%)	<0.01
**All-cause death**	111(18.62%)	39(13.36%)	72(23.68%)	<0.01
**Stroke**	67(11.24%)	11(3.77%)	56(18.42%)	<0.01
**MI**	63(10.57%)	14(4.79%)	49(16.12%)	<0.01
**Emergent coronary revascularization**	40(6.71%)	5(1.71%)	35(11.51%)	<0.01

TyG, triglyceride-glucose; MACCE, major adverse cardiovascular and cerebrovascular event; MI, myocardial infarction.

**Figure 3:**
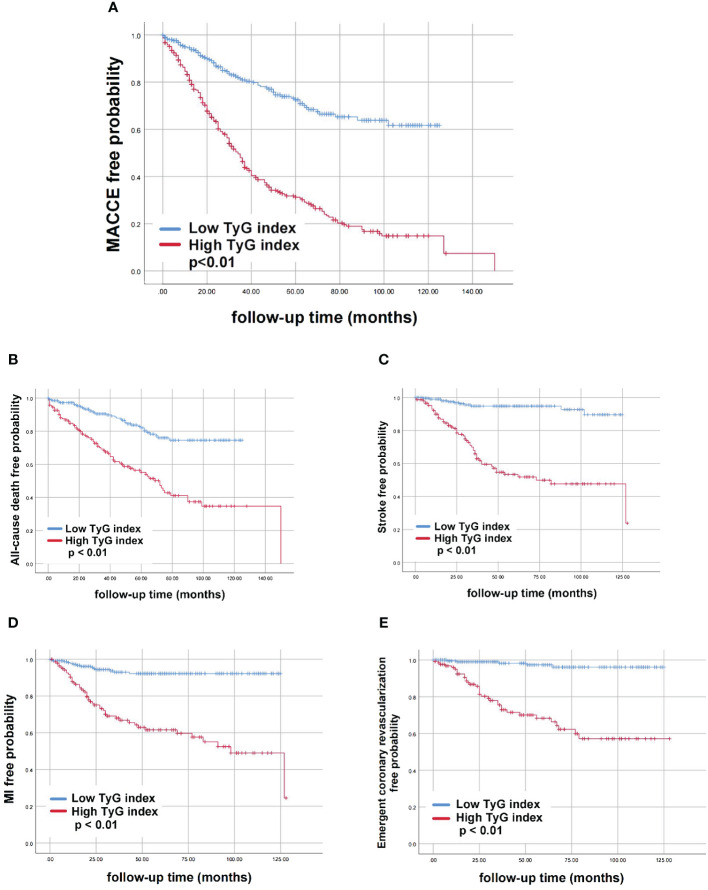
Kaplan-Meier curves for different groups of endpoint events. **(A)** MACCE; **(B)** All-cause death; **(C)** Stroke; **(D)** MI; **(E)** Emergent coronary revascularization. AUC; TyG, triglyceride-glucose; TyG, triglyceride-glucose; MACCE, major adverse cardiovascular and cerebrovascular event; MI, myocardial infarction.

According to Kaplan-Meier survival analysis, patients with a high TyG index exhibited an increased risk of MACCE (*P*<0.01). In the high TyG index group, there was a significantly higher occurrence of all-cause death, stroke, MI, and emergent coronary revascularization compared to the low TyG index group (*P*<0.01) (**Figure 3**).

To further assess the impact of the TyG index on MACCE, we used four distinct Cox proportional risk models incorporating different categories of confounders for separate analyses. The result revealed that as a continuous variable, the TyG index was independently associated with an elevated risk of MACCE for each unit increase in the index (Model 1: 2.27, 1.90–2.70, *P*<0.01; Model 2: 2.26, 1.85–2.75, *P*<0.01; Model 3: 2.44, 1.89–3.16 *P*<0.01; Model 4: 2.09, 1.59–2.74, *P*<0.01) (**Table 4**).

**Table 4:** Predictive value of TyG index for MACCE in different Cox proportional risk models.

	TyG index as a continuous variable^a^			TyG index as a categorical variable^b^		
	HR	95% CI	*P* value	HR	95% CI	*P* value
**Crude model**	2.18	1.85–2.58	<0.01	3.69	2.80–4.84	<0.01
**Model 1**	2.27	1.90–2.70	<0.01	3.84	2.90–5.08	<0.01
**Model 2**	2.26	1.85–2.75	<0.01	3.74	2.79–5.01	<0.01
**Model 3**	2.44	1.89–3.16	<0.01	3.56	2.60–4.90	<0.01
**Model 4**	2.09	1.59–2.74	<0.01	3.01	2.16–4.21	<0.01

Model 1 Age, sex, BMI, smoking, SBP, DBP.

Model 2 Add to Model 1: Hypertension, T2DM, Dyslipidemia, Prior CVDs, Prior PCI, Prior stroke, Family history of CVDs.

Model 3 Add to Model 2 the variable for clinical lab results: TC, HDL-C, LDL-C, HbA1c.

Model 4 Add to Model 3 the variable for clinical medication: Antiplatelet medication, ACEI/ARB, CCB, β-Blocker, Antidiabetic agents, Statins, Antidepressants, Benzodiazepines.

a. The HR was assessed with each 1-unit increase in the TyG index.

b. The HR was examined with the low TyG index group as the reference.

When the TyG index was examined as a categorical variable, a distinct correlation could also be observed between the high TyG index group and MACCE (Model 1: 3.84, 2.90–5.08, *P*<0.01; Model 2: 3.74, 2.79–5.01, *P*<0.01; Model 3: 3.56, 2.60–4.90, *P*<0.01; Model 4: 3.01, 2.16–4.21, *P*<0.01).

We examined the prognostic significance of the TyG index for all outcome events through both univariate and multivariate analyses. The results showed that the TyG index was independently associated with a high risk of MACCE when used as a continuous variable (2.09, 1.59–2.74, *P*<0.01), all-cause death (2.55, 1.65–3.93, *P*<0.01), stroke (2.79, 1.50–5.16, *P*<0.01),MI (2.54, 1.40–4.60, *P*<0.01), emergent coronary revascularization (4.37, 1.94–9.86, *P*<0.01). As a categorical variable, the TyG index could still serve as an independent predictor of both MACCE(3.01, 2.16–4.21, *P*<0.01) and all-cause death(2.99, 1.81–4.95, *P*<0.01), stroke(6.14, 2.86–13.15, *P*<0.01), MI(7.38, 3.36–16.18, *P*<0.01), or emergent coronary revascularization(10.10, 3.26–31.27, *P*<0.01) (**Table 5**).

**Table 5:** Predictive value of the TyG index for endpoint events in univariate and multivariate analyses.

	Univariate analysis			Multivariate analysis^a^		
	HR	95% CI	*P* value	HR	95% CI	*P* value
TyG index as a continuous variable^b^						
** MACCE**	2.18	1.85–2.58	<0.01	2.09	1.59–2.74	<0.01
** All-cause death**	2.30	1.77–2.98	<0.01	2.55	1.65–3.93	<0.01
** Stroke**	3.16	2.31–4.33	<0.01	2.79	1.50–5.16	<0.01
** MI**	2.61	1.90–3.59	<0.01	2.54	1.40–4.60	<0.01
** Emergent coronary revascularization**	3.58	2.46–5.22	<0.01	4.37	1.94–9.86	<0.01
TyG index as a categorical variable^c^						
** MACCE**	3.69	2.80–4.84	<0.01	3.01	2.16–4.21	<0.01
** All-cause death**	3.35	2.26–4.95	<0.01	2.99	1.81–4.95	<0.01
** Stroke**	9.15	4.78–17.50	<0.01	6.14	2.86–13.15	<0.01
** MI**	6.40	3.53–11.62	<0.01	7.38	3.36–16.18	<0.01
** Emergent coronary revascularization**	13.99	5.48–35.74	<0.01	10.10	3.26–31.27	<0.01

a. The variable factors included in the multifactor analysis are the same as in Model 4; b. The HR was assessed with each 1-unit increase in the TyG index; c. The HR was examined with the low TyG index group as the reference. TyG, triglyceride-glucose; MACCE, major adverse cardiovascular and cerebrovascular event; MI, myocardial infarction.

**Figure 4:**
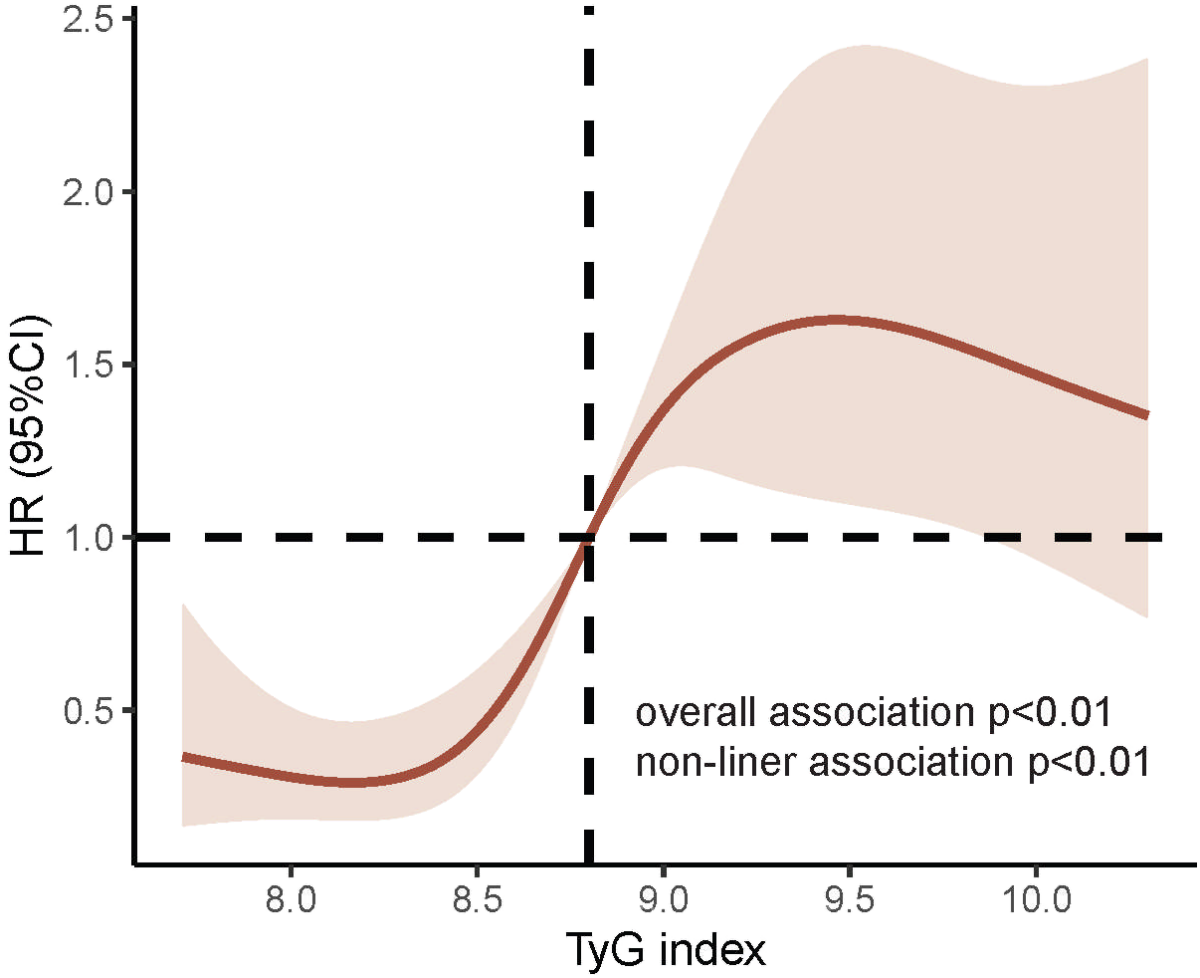
RCS curves for TyG index associated with MACCE. *P* value for nonlinear association<0.01; *P* value for overall association<0.01; the red line represents the references for HR, and the pink area indicates the 95% CI. RCS analysis was adjusted according to Model 4. RCS, Restricted cubic splines; TyG, triglyceride-glucose; MACCE, major adverse cardiovascular and cerebrovascular event; MI, myocardial infarction.

The multivariate RCS curves generated according to Model 4 showed a nonlinear relationship between the TyG index and MACCE (nonlinear *P*<0.01, overall *P*<0.01). The HR value for distinguishing the presence of MACCE was closest to 1 when the TyG index was 8.80 (**Figure 4**).

### Subgroup analysis

**Figure 5:**
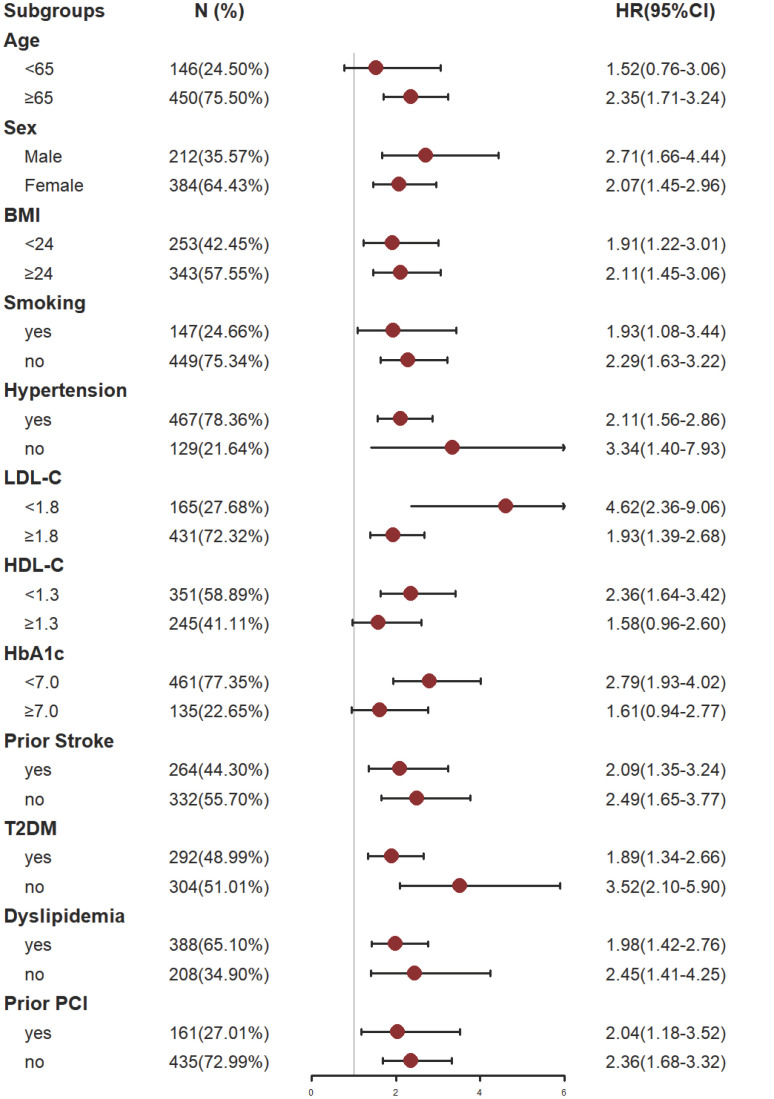
Subgroup analysis of the effect of the TyG index on MACCE. The gray vertical line represents an HR of 1. Subgroup analysis using Model 4. BMI, body mass index; LDL-C, low density lipoprotein cholesterol; HDL-C, high-density lipoprotein cholesterol; HbA1C, glycosylated hemoglobin A1c; T2DM, type 2 diabetes mellitus; PCI, percutaneous coronary intervention.

The study participants were divided into subgroups based on age (<65 or ≥65 years); male sex(yes or no), BMI(< 24 or ≥ 24 kg/m^2^), Somking(yes or no), Hypertension(yes or no), LDL-C(<1.8 or ≥1.8 mmol/L), HDL-C(<1.3 or ≥1.3 mmol/L), HbA1C(<7 or ≥7%), Prior stroke(yes or no), T2DM(yes or no), Dyslipidemia(yes or no) and Prior PCI (yes or no) for the purposes of further validating the TyG index’s ability to predict MACCE in different subgroups (**Figure 5**).

The analysis results showed that TyG index has a good predictive effect on the prognosis of most patients with CHD complicated with depression. However, the predictive value of TyG index for MACCE in patients under 65 years old, smokers, patients with HDL-C ≥ 1.3 mmol/l and patients with HbA1c ≥ 7.0% had some limitations. The reasons might be as follows: patients who smoke often suffer from respiratory diseases; While patients with HbA1c ≥ 7.0% had poor long-term blood glucose control; These influencing factors might have adverse effects on the prognosis of patients with coronary heart disease complicated with depression. For patients under 65 years old or with HDL-C ≥ 1.3 mmol/l, their physical condition is relatively better, and the possibility of MACCE is lower. Even if there is metabolic abnormality, it is usually easier to be corrected, so it might be difficult to predict such patients through a single TyG index to some extent. For these people, it might be possible to make better prognosis prediction by combining TyG index with other influencing factors.

### TyG index predicts the incremental effect of MACCE

The initial risk model of baseline incorporated diverse factors, including age, sex, BMI, SBP, DBP, smoking, TC, HDL-C, LDL-C, HbA1c, hypertension, T2DM, dyslipidemia, prior CVDs, prior PCI, prior stroke, family history of CVDs, and the use of antiplatelet medication, ACEI/ARB, CCB, β-Blocker, antidiabetic agents, statins, antidepressants, benzodiazepines. Upon integration into the baseline risk model, the TyG index significantly enhanced the precision of MACCE prediction, reclassification and discrimination, in comparison to TG or FBG alone (NRI: 0.028, 0.001–0.049; IDI:0.145, 0.011–0.193; C-Index: 0.698, 0.667–0.729; all *P*<0.01) (**Table 6**).

**Table 6:** Incremental effect of TyG index and its components for predicting MACCE.

	NRI	95%CI	*P* value	IDI	95%CI	*P* value	C-Index	95%CI	*P* value
**Baseline risk model**	REF			REF			0.672	0.640–0.705	<0.01
**+TG**	0.006	0–0.022	<0.01	0.069	-0.027–0.168	0.545	0.677	0.645–0.709	<0.01
**+FBG**	0.007	0–0.018	0.182	0.075	0.005–0.149	<0.01	0.687	0.655–0.719	<0.01
**+TyG index**	0.028	0.001–0.049	<0.01	0.145	0.011–0.193	<0.01	0.698	0.667–0.729	<0.01

NRI, net reclassification improvement; IDI, integrated discrimination improvement; TG, triglyceride; FBG, fasting blood; TyG, triglyceride-glucose.

## Discussion

TyG index is a surrogate marker of IR and metabolic abnormalities, which is closely related to the prognosis of a variety of diseases. Over the past few years, numerous studies have validated the strong correlation between the TyG index score and the occurrence and outcome of CVD. Nonetheless, the predictive significance of the TyG index in individuals with CHD complicated with depression is still uncertain. Our study showed that (1) The elevation of the TyG index was closely correlated with an increased risk of adverse events in patients with CHD complicated with depression. (2) The TyG index exhibited an independent association with the risk of all-cause death, stroke, MI, and emergency coronary revascularization, both as continuous and categorical variables. (3) In patients with both CHD and depression, there existed a nonlinear relationship between the TyG index and the risk of MACCE, with the optimal cut-off value for predicting MACCE determined as 8.80.

Depression can lead to autonomic dysfunction and endocrine imbalance, exerting adverse effects on CVD through various mechanisms (27, 28). Specifically, depression induces dysregulation of the sympathetic–adrenomedullary system, which leads to the secretion of catecholamines, resulting in increased heart rate, blood pressure, and myocardial contraction, leading to increased cardiac load and risk of coronary artery spasm (4, 29, 30). In addition, depression leads to neuroendocrine disorders by activating the hypothalamic–pituitary–adrenal (HPA) axis and promoting the secretion of corticotropin-releasing hormone, which in turn promotes the secretion of adrenocorticotropic hormone, resulting in increased cortisol levels, abnormal cortisol rhythms, and fluctuating blood glucose (31).

Inflammation is a recognized risk factor for atherosclerosis and CHD (32). Depression can result in elevated levels of C-reactive protein, interleukin (IL)-1β, IL-6, and tissue necrotic factor-alpha, as well as an increase in the concentration of inflammatory molecules, such as NLRP3 inflammasomes (33, 34). It can also lead to an increase in oxidative stress markers (35), which in turn promote plaque formation and rupture, affect thrombosis formation, and have adverse effects on CV health (36).

Depression also leads to disorders of lipid metabolism. Existing evidence suggests that high TC and LDL levels are closely associated with depressive symptoms, as well as severity and the expected course of depression (37). Similarly, depression can contribute to dyslipidemia and MetS, thereby affecting the development of CVD (38). In addition, depression can also induce an increase in platelet reactivity and secretion through the platelet-serotonin pathway and platelet adenosine response (39, 40). This further increases the susceptibility of patients with depression to acute thrombotic events and ischemic heart disease as well as the risk of mortality after MI (38). It can be seen that depression can make patients with cardiovascular disease more vulnerable, have a worse prognosis and a higher risk of cardiovascular events through a variety of ways (33).

The TyG index, a marker of IR, is strongly associated with not only the risk of adverse events in CHD but also depression. Individuals with higher TyG index are significantly more likely to experience depressive symptoms (21), and elevated IR and TyG index exacerbate depression and reduce the efficacy of antidepressant therapies (41, 42). On the other hand, depression can also lead to the aggravation of IR severity and the increase of TyG index (41) and TyG index (43). The specific mechanisms underlying this association may be related to altered dopamine signaling, 5-hydroxytryptaminergic transmission, the HPA axis, neurogenesis, neuroinflammation, opioid-mediated pathways, gut microbiome, and gut-brain signaling (41).

More importantly, IR, as characterized by the TyG index, can have a serious negative impact on the outcome of CHD comorbid with depression. Severe IR induces imbalances in glucose and lipid metabolism and elevates the TyG index while exacerbating the inflammatory response in the body (14, 44, 45); this further damages the already fragile vascular endothelium of this special population and induces atherosclerosis, as well as progression and rupture of coronary plaques (44). Additionally, it induces alterations in the fibrinolytic system and disrupts the balance of coagulation, leading to thrombosis (46). Persistent IR increases sympathetic nervous system activity, leading to blood pressure fluctuations and retention of water and sodium, thereby increasing cardiac load (47). It can also further affect the prognosis of patients with CHD complicated with depression by inducing high glycosylation to promote myocardial fibrosis (48). Our study confirmed that TyG, as a marker of IR, is an independent risk factor for MACCE in patients with CHD complicated with depression, especially when the TyG score is >8.80. Special attention should be paid to such patients in clinical practice.

Adopting a healthy lifestyle and diet is beneficial for improving IR, reducing the TyG index score, and promoting recovery of psychological health in patients with depression (49). Studies have demonstrated that exercise (50), vitamin supplementation, healthy diet, sun exposure, and sleep hygiene can effectively improve IR and depression (51). Therefore, a healthy lifestyle and diet are particularly important for patients with concomitant CHD and depression. Furthermore, this specific patient group should be regularly tested for blood glucose and lipids levels (52), for timely detection of changes in TyG levels, allowing for early interventions to prevent the occurrence of adverse events.

This study had some limitations. First, this is a single-center, retrospective, cohort study from China, which may have some external validity issues due to differences in culture, population, region, and healthcare system. Second, the TyG index included in this study was derived from a single calculation and failed to be studied with dynamically changing TyG index data. Third, the degree of patient depression was not accurately captured in this retrospective study, and future studies could further stratify patients with different levels of depression. Nevertheless, this study has clinical value as the first study to evaluate the prognostic factors of the TyG index in patients with CHD complicated with depression.

## Conclusions

TyG index is highly correlated with the risk of MACCE in patients with CHD complicated with depression and may be a predictor of MACCE in this high-risk group, playing an important role in risk stratification and clinical management. The TyG index has an important prognostic value for patients with CHD complicated with depression. In the future, more in-depth prospective clinical studies can be conducted to further clarify the impact of the TyG index on endpoint events in this special population.

## Data availability statement

The raw data supporting the conclusions of this article will be made available by the authors, without undue reservation.

## Ethics statement

The studies involving humans were approved by Clinical Research Ethics Committee of the Dongfang Hospital of Beijing University of Chinese Medicine. The studies were conducted in accordance with the local legislation and institutional requirements. The ethics committee/institutional review board waived the requirement of written informed consent for participation from the participants or the participants’ legal guardians/next of kin because this is a retrospective study. The data of all patients have been anonymous, so the written informed consent was waived.

## Author contributions

WZ: Data curation, Formal analysis, Project administration, Writing – original draft, Conceptualization. JW: Data curation, Investigation, Writing – review & editing. DC: Formal analysis, Methodology, Writing – review & editing, Conceptualization. WD: Validation, Writing – review & editing. JH: Resources, Supervision, Writing – review & editing. YG: Investigation, Writing – review & editing. YL: Validation, Visualization, Writing – review & editing. RL: Investigation, Writing – review & editing. XL: Writing – review & editing, Project administration. ZS: Supervision, Writing – review & editing. HZ: Funding acquisition, Project administration, Resources, Writing – review & editing.
