# Supplementary material for: Triglyceride-glucose index as a potential predictor of major adverse cardiovascular and cerebrovascular events in patients with coronary heart disease complicated with depression
Source: Front Endocrinol (Lausanne). 2024 Jun 26;15:1416530. doi: 10.3389/fendo.2024.1416530 (PMC11240118; doi:10.3389/fendo.2024.1416530)
Supplement: Supplementary file 1 [file Table_1.docx]

**Table. S1 Analysis of the correlation between TyG index and cardiovascular risk factors**

| Variable | Correlation coefficient | *P* value |
| --- | --- | --- |
| Age | -0.082 | 0.046 |
| Male | -0.092 | 0.025 |
| BMI | 0.199 | <0.01 |
| Somking | -0.011 | 0.784 |
| SBP | -0.011 | 0.786 |
| DBP | 0.004 | 0.926 |
| TC | 0.337 | <0.01 |
| HDL-C | -0.244 | <0.01 |
| LDL-C | 0.271 | <0.01 |
| HbA1c | 0.474 | <0.01 |
| Family history of CVDs | 0.051 | 0.212 |
| Hypertension | 0.041 | 0.319 |
| T2DM n | 0.382 | <0.01 |
| Dyslipidemia | 0.056 | 0.169 |
| Prior CVDs | -0.021 | 0.616 |
| Prior PCI | 0.030 | 0.462 |
| Prior stroke | 0.058 | 0.155 |

BMI, body mass index; SBP, systolic blood pressure; DBP, diastolic blood pressure; TC, total cholesterol; TG, triglyceride; LDL-C, low-density lipoprotein cholesterol; HDL-C, high-density lipoprotein cholesterol; FBG, fasting blood glucose; HbA1c, glycosylated hemoglobin; TyG, triglyceride-glucose;T2DM, diabetes mellitus type 2; CVD, cardiovascular disease; PCI, percutaneous coronary intervention; ACEI, angiotensin converting enzyme inhibitor; ARB, angiotensin receptor blocker; CCB, calcium channel blockers.
